# Exploiting whole genome sequence data to fine map and characterize candidate genes within a quantitative trait loci region affecting androstenone on porcine chromosome 5

**DOI:** 10.1111/age.12615

**Published:** 2017-10-16

**Authors:** M. van Son, R. Agarwal, M. P. Kent, H. Grove, E. Grindflek, S. Lien

**Affiliations:** ^1^ Topigs Norsvin Storhamargata 44 2317 Hamar Norway; ^2^ Department of Animal and Aquacultural Sciences (IHA) Faculty of Life Sciences (BIOVIT) Centre for Integrative Genetics (CIGENE) Norwegian University of Life Sciences (NMBU) PO Box 5003 1432 Ås Norway

**Keywords:** boar taint, fine mapping, pig, whole genome resequencing

## Abstract

Male piglets are routinely castrated to eliminate boar taint. However, this treatment is undesirable, and alternative approaches, including genetic strategies to reduce boar taint, are demanded. Androstenone is one of the causative agents of boar taint, and a QTL region affecting this pheromone has previously been reported on SSC5: 22.6–24.8 Mb in Duroc. The QTL region is one of the few reported for androstenone that does not simultaneously affect levels of other sex steroids. The main objective of this study was to fine map this QTL. Whole genome sequence data from 23 Norwegian Duroc boars were analyzed to detect new polymorphisms within the QTL region. A subset of 161 SNPs was genotyped in 834 Duroc sires and analyzed for association with androstenone in adipose tissue and testosterone, estrone sulphate and 17*β*‐estradiol in blood plasma. Our results revealed 100 SNPs significantly associated with androstenone levels in fat (*P *<* *0.001) with 94 of the SNPs being in strong linkage disequilibrium in the region 23.03–24.27 Mb. This haplotype block contains at least four positional candidate genes (*HSD17B6*,*SDR9C7*,*RDH16* and *STAT6*) involved in androstenone biosynthesis. No significant associations were found between any of the SNPs and levels of testosterone and estrogens, confirming previous findings. The amount of phenotypic variance explained by single SNPs within the haplotype block was as high as 5.4%. As the SNPs in this region significantly affect levels of androstenone without affecting levels of other sex steroids, they are especially interesting as genetic markers for selection against boar taint.

## Introduction

Surgical castration of male piglets has a long tradition in European pig production where it is used to reduce boar taint in meat from boars and thereby strengthen consumer's acceptability for pork meat. However, this practice is controversial, and discussions are ongoing which could lead to a ban in Norway and the EU due to animal welfare concerns (Bee *et al*. 2015). Castration is also undesirable because it suppresses levels of testicular steroids, thereby inhibiting growth rate, feed efficiency and lean tissue growth (Xue *et al*. 1997). Alternative solutions, such as genetic selection strategies, are therefore being pursued to reduce accumulation of the main components of boar taint—androstenone and skatole—in adipose tissue (Merks *et al*. 2009).

Androstenone (5*α*‐androst‐16‐en‐3‐one) is a 16‐androstene steroid produced by the Leydig cells of the testis along with other sexual steroids including testosterone and estrogens (Brooks & Pearson 1986), whereas skatole (3‐methylindole) is a catabolic product of tryptophan generated by intestinal bacteria (Yokoyama & Carlson 1974). The heritability is high for both androstenone and skatole, varying from 0.47–0.67 and 0.37–0.41 respectively (Sellier *et al*. 2000; Grindflek *et al*. 2011b; Windig *et al*. 2012). Several genomic studies have identified quantitative trait loci (QTL) (e.g. Grindflek *et al*. 2011a,b; Duijvesteijn *et al*. 2014; Rowe *et al*. 2014; Große‐Brinkhaus *et al*. 2015) and/or candidate genes (Zadinová *et al*. 2016) affecting levels of androstenone and skatole.

Using a combined approach of a genome‐wide association study and linkage disequilibrium–linkage analysis, Grindflek *et al*. (2011a) reported a highly significant QTL on SSC5 for androstenone levels in fat of Norwegian Duroc pigs. The QTL is an attractive candidate for selective breeding because of its potential to reduce levels of androstenone without affecting the important sex steroids testosterone, estrone sulphate and 17*β*‐estradiol (Grindflek *et al*. 2011a). The most likely position of the QTL was located at 22–25 Mb on SSC5 (*Sus scrofa* genome build 10.2), a region containing at least six positional candidate genes: *retinol dehydrogenase 5* (*RDH5*)*, sulfite oxidase* (*SUOX*), *hydroxysteroid 17 beta hydroxysteroid dehydrogenase 6* (*HSD17B6*), *short chain dehydrogenase/reductase family 9C member 7* (*SDR9C7*)*, retinol dehydrogenase 16 (all‐trans)* (*RDH16*), and *signal transducer and activator of transcription 6* (*STAT6*). The objective of this study was to fine map this QTL by increasing the marker density within the region, scrutinize positional candidate genes for polymorphisms and, if possible, disclose underlying functional variants associated with androstenone levels in entire male pigs.

## Material and methods

### Animals and phenotypes

A total of 925 Duroc boars from 70 paternal half‐sib families (6–27 offspring each), reared under the same conditions using standard commercial feed until reaching slaughter weight (100 kg; on average 156 days) were included in this study. All animals were cared for according to laws, internationally recognized guidelines and regulations controlling experiments with live animals in Norway according to the rules given by the Norwegian Animal Research Authority (The Animal Protection Act of December 20th, 1974, and the Animal Protection Ordinance Concerning Experiments with Animals of January 15th, 1996). Blood samples were collected before slaughter for plasma suspension and DNA extraction, whereas subcutaneous adipose tissue was collected from the neck 20–30 min post‐slaughter for measurement of androstenone. Levels of testosterone, estrone sulphate and 17*β*‐estradiol were also measured from plasma from the same individuals. For a detailed description of the materials, see Grindflek *et al*. (2011a).

### Whole genome sequencing and detection of single nucleotide polymorphisms

A subset of 23 Duroc boars were prioritized for individual whole genome sequencing on Illumina GAII according to manufacturer's protocols. The boars were frequently used as AI boars during the years of 2010 to 2013, and two of them overlapped with the genotyped boars whereas the others were relatives. The reads were paired‐end (2 × 100 bp) reads and were quality checked using fastqc version 0.10.1 (Babraham Bioinformatics) before being processed using a custom Perl script to remove duplicated reads, trim sequencing primer sequence and any flanking 3′ bases and remove reads shorter than 80 nucleotides. This removed approximately 15% of the reads. A total of 4.9 billion reads remained after filtering with an average per‐animal coverage ranging from 9–17× at an overall per‐base quality >30. Of these remaining reads, 77% were successfully mapped against *S. scrofa* genome build 10.2 using bowtie2 (version 2.0.0) with default parameters (Langmead & Salzberg 2012).

After mapping, 64 117 single nucleotide polymorphisms (SNPs) were detected within the QTL region using freebayes (Garrison & Marth 2012). SNPs located within repeated regions, as defined by pig Ensembl release 67, were excluded before applying stringent filtering criteria to reduce incidences of false positive SNPs: (i) all genotypes for each of the 23 Duroc samples supported by at least two reads and (ii) each SNP having at least one homozygous and one heterozygous genotype among the 23 boars to be selected for genotyping. Altogether, 1545 SNPs passed this filtering, and 1488 of these were classified in relation to protein‐coding genes in the region using variant effect predictor (VEP) (McLaren *et al*. 2016) for *S. scrofa* genome build 10.2 (www.ensembl.org/Sus_scrofa/Tools/VEP). Briefly, 77 SNPs were defined as upstream (i.e. <1000 bp from exon 1 start), 144 SNPs were described as being within coding regions (including exons and untranslated regions), 715 SNPs as intronic and 552 SNPs as intergenic. Of the 144 exonic SNPs, 26 were predicted to be non‐synonymous. The effect of amino acid changes on protein function were predicted using SIFT (Kumar *et al*. 2009).

A subset of 185 SNPs were selected for genotyping based on allele frequencies estimated from the sequencing data, their location within positional candidate genes and simultaneously ensuring a broad distribution of SNPs within the QTL region. Genotyping assays were designed using the massarray assay design software (Sequenom) resulting in multiplexing levels from 31 to 40 markers. The SNPs were genotyped with the iPLEX protocol according to the manufacturer's instructions (Sequenom). massarray typer software was used for automated genotype calling, but clusters were also manually inspected. SNPs displaying low minor allele frequency (<0.01) and/or showing less than 10% missing data were discarded from further analysis. Animals lacking androstenone recordings were removed from the dataset, leaving 161 SNPs genotyped in 834 boars for the association analysis. Genotypes were also retrieved for porcine 60K Illumina BeadChip SNPs in this QTL region for the same boars, altogether 21 SNPs, as available from Grindflek *et al*. (2011a). A detailed description of the data are given in Table S1. The newly identified SNPs have been deposited at NCBI dbSNP and their reference SNP (rs) numbers can be found in Table S1.

### Association mapping

beagle version 3.3.1 software was used to infer phase and impute missing genotypes in the dataset (Browning & Browning 2007). haploview was used to estimate pair‐wise linkage disequilibrium (LD) between SNP positions and identify haplotypes (Barrett *et al*. 2005). The association study was performed using asreml software to determine SNP and haplotype effects (Gilmour *et al*. 2001). Relationships among individuals were accounted for using a pedigree‐based relationship matrix.

The statistical mixed model was as follows:

$$Y=\text{sire}+\text{herd‐year‐season}+\text{waiting‐station}+\text{pen}+\text{animal}+\text{sample‐date}+\text{SNP}+\text{age}\_25\;\text{kg}+\text{days‐test}+\text{days‐wait}+\text{liveborn}+{\left(\text{liveborn}\right)}^{2}+e,$$

where *Y *= ln (androstenone), where androstenone is μg/g levels of androstenone in adipose tissue. Levels of testosterone, estrone sulphate and 17*β*‐estradiol in plasma were analyzed with the same model. Fitted fixed effects were sire, herd‐year‐season, waiting in boar test station before slaughter or not (waiting‐station) and pen. Covariates included were age at 25 kg (age_25 kg, start of the boar test), days in the boar test (days‐test), days in waiting station (days‐wait, end of boar test to slaughter) and number of live born in the same litter (liveborn). Random effects fitted were individual ID, sample date for adipose tissue or plasma and SNP genotype or haplotype.

The likelihood ratio test (LRT) was used to test for SNP association. LRT scores were calculated as two times the ratio between the difference in log‐likelihood (LogL) values between the model with SNP or haplotype effects and the model without these effects. LRT scores were assumed to be chi‐squared distributed with one degree of freedom. LRT values corresponding to a *P*‐value of 0.001 were considered significant. The significance threshold was adjusted to account for the multiple tests performed, using the effective number of independent tests (Gao *et al*. 2008). The adjusted significance level for LRT values was 18.3. A two sample *t*‐test was used to test the difference in phenotypic means between boars homozygous for the most common haplotype and the remaining boars.

## Results and discussion

**Figure 1:**
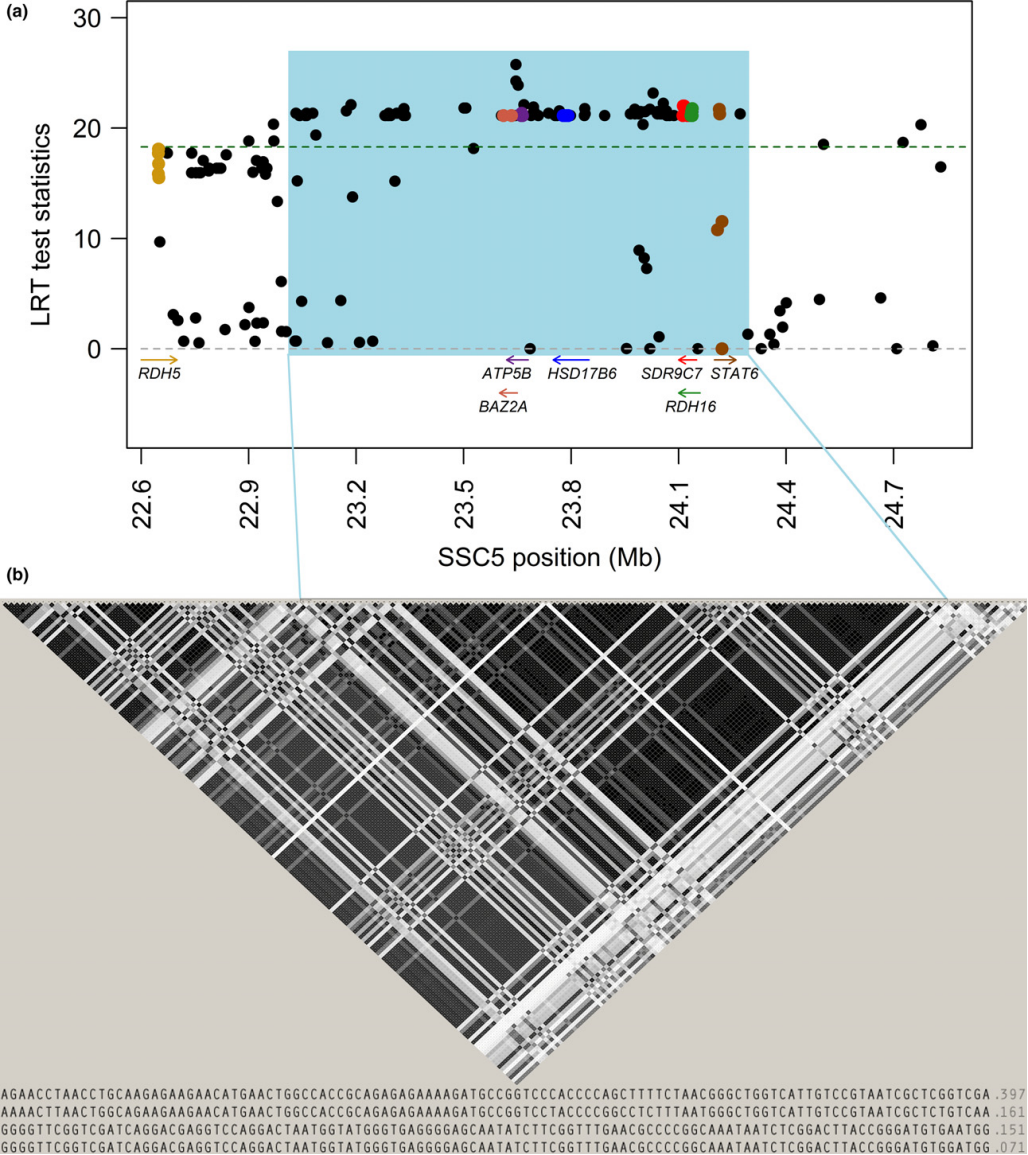
(a) Likelihood‐ratio test (LRT) scores from single‐marker association mapping between single nucleotide polymorphisms (SNPs) and androstenone levels in fat of Duroc pigs. On the *x*‐axis are the physical coordinates of the SNPs based on *Sus scrofa* build 10.2 and candidate genes in the region. On the *y*‐axis is significance threshold for SNPs with a dashed line at LRT score 18.3 (multiple testing adjusted *P*‐value of 0.001). (b) Linkage disequilibrium plot for the QTL region on SSC5, with the haplotype block at position 23.03–24.27 Mb in blue. The haplotypes within the haplotype block and their frequencies are under the plot.

Previous QTL studies in Norwegian pig breeds using the 7K and 60K Porcine SNP arrays have identified several QTLs underlying androstenone, skatole and sex hormones related to reproduction (Grindflek *et al*. 2011a,b). One of the most interesting QTLs was detected at position 22.6–24.8 Mb on SSC5 (*S. scrofa* genome build 10.2) in the Norwegian Duroc breed. This highly significant region for levels of androstenone does not affect levels of testosterone or estrogens, which makes it particularly interesting with respect to implementation in breeding schemes (Grindflek *et al*. 2011a). Support for a QTL in this position on SSC5 was found in Danish Landrace boars with the most significant results detected for two SNPs (*H3GA0016037* and *ASGA002509*) mapping 4 Mb apart at position 20.90–24.35 Mb (Rowe *et al*. 2014). In an attempt to fine map this QTL, and possibly disclose putative causal variation within candidate genes in the region, we selected SNPs from whole genome sequence data from 23 Duroc boars and genotyped them in 834 boars with recordings on androstenone, testosterone, estrone sulphate and 17*β*‐estradiol. Association analyses of the SNPs obtained from the sequences revealed that as many as 100 out of the selected SNPs from the SSC5 QTL region were significantly associated with levels of androstenone in adipose tissue (*P *<* *0.001/multiple testing adjusted LRT = 18.3), reflecting the generally high LD in the region (Fig. 1 & Table S1).

**Figure 2:**
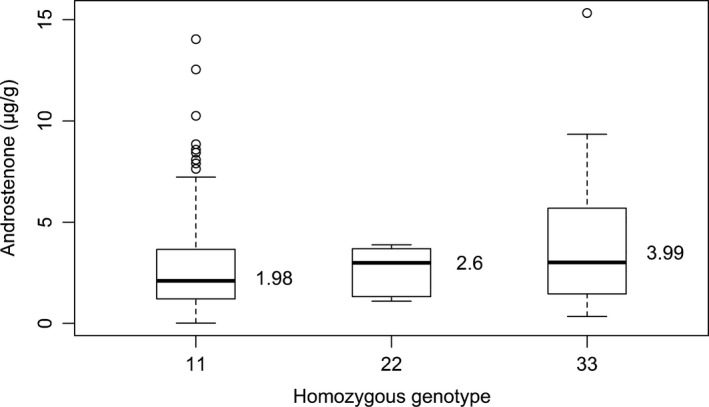
Boxplots of the distribution of androstenone levels for the haplotype block on SSC5 in Duroc pigs. On the *x*‐axis are the homozygous haplotypes of the three most common haplotypes. The mean for each homozygous haplotype is given beside the boxes.

The most significant results were found for two SNPs (*ASGA0025083* and *rs329732704*), which were in high LD with each other (*r* ^2^ = 0.98) and physically linked (only 6 kb apart) at position 23.65 Mb. This location is within the intergenic region between the *bromodomain adjacent to zinc finger domain 2A* (*BAZ2A*) and *ATP synthase, H+ transporting, mitochondrial F1 complex, beta polypeptide* (*ATP5B*) genes. However, based on the available literature, these genes do not seem to have an obvious biological function related to androstenone. With this in mind, it might be more likely that the QTL effect is caused by variation within one of the many candidate genes located in close vicinity of these two genes (Rowe *et al*. 2014). However, most of the SNPs significantly associated with androstenone (94 out of the 100 SNPs) are located within an extended haplotype block of 1.24 Mb at 23.03–24.27 Mb (Fig. 1), suggesting that this is the most likely region affecting the trait. Construction of haplotypes across this block identified four haplotypes with frequencies above 5% (Fig. 1b). The two most frequent haplotypes within this block are associated with low levels of androstenone, whereas the other two are associated with high levels. Mean levels of androstenone for animals homozygous for haplotype 1 (freq. 0.4) and haplotype 2 (freq. 0.16) are 1.98 μg/g (±2.56) and 2.61 μg/g (±1.17) respectively (Fig. 2). Mean levels of androstenone for animals homozygous for haplotype 3 (freq. 0.15) is 3.99 μg/g (±3.41). Only two animals were homozygous for haplotype 4, and their androstenone levels were 0.77 and 2.76 μg/g respectively. Boars homozygous for haplotype 1 had significantly lower levels of androstenone than did the remaining boars. The individual SNPs within this block explained up to 5.4% of the phenotypic variance, which is similar to the results observed by Rowe *et al*. (2014).

The extended haplotype block contains at least four candidate genes likely involved in biochemical pathways affecting androstenone levels: *HSD17B6, SDR9C7, RDH16* and *STAT6*. Notably, the haplotype block at 23.03–24.27 Mb does not contain the *RDH5* and *SUOX* genes, which thereby excludes these as positional candidate genes for the QTL. The *HSD17B6* gene, located at position 23.78–23.80 Mb, has previously been reported as an obvious candidate gene for androstenone (Grindflek *et al*. 2011a) due to its a multifunctional role in androgen biosynthesis and metabolism (Moeller & Adamski 2009). More specifically, the 17*β*‐hydroxysteroid dehydrogenases are involved in the activation and inactivation of steroid hormones by catalyzing redox reactions at position C17 of the steroid ring structure. The *HSD17B6* gene was found to be associated with androstenone through pathway analysis (Sahadevan *et al*. 2014) and has been suggested as a positional candidate gene for androstenone in this QTL region by Rowe *et al*. (2014) and Grindflek *et al*. (2011a). Moreover, several other members of the HSD17B gene family have previously been found to be differentially expressed in Norwegian Duroc boars with extreme levels of androstenone (Moe *et al*. 2007, 2008). The *SDR9C7* gene, located at position 24.11–24.13 Mb, belongs to the SDR superfamily, comprising genes that are involved in oxidoreduction reactions of many substrates, including steroid hormones (Kowalik *et al*. 2009). However, dehydrogenase activity of SDR9C7 with steroid substrates has not been shown, and its exact functional role remains unknown (Kowalik *et al*. 2009). The *RDH16* gene, located at position 24.13–24.14 Mb, encodes an enzyme that oxidizes 3*α*‐hydroxysteroids such as androstanediol and androsterone to dihydrotestosterone and androstanedione (Gough *et al*. 1998). In pigs, androstenone is catabolized to 3*α*‐androstenol and 3*β*‐androstenol by enzymes with 3*α*‐ and 3*β* hydroxysteroid dehydrogenase activities (Brooks & Pearson 1986). If *RDH16* is able to reduce androstenone by its 3*α*‐hydroxysteroid dehydrogenase activity, it is a very interesting candidate gene. The *STAT6* gene, located at position 24.21–24.22 Mb, encodes a transcription factor that is activated by cytokines and growth factors (Leonard 2001). For example STAT6 is involved in the regulation of the *hydroxy‐delta‐5‐steroid dehydrogenase, 3 beta* (*HSD3B*) promoter (Simard *et al*. 2005). HSD3B dehydrogenase activity is required for the biosynthesis of active estrogens and androgens from progesterone (Simard *et al*. 2005) and for the metabolism of androstenone to 3*β*‐androstenol, as mentioned above. Moreover, a *STAT6* binding site has been identified in the *cytochrome P450 family 2 subfamily E member 1* (*CYP2E1*) gene (Wang *et al*. 2010), which is important for both androstenone and skatole metabolism (Squires & Lundström 1997; Doran *et al*. 2002; Moe *et al*. 2009).

Two missense mutations highly associated with androstenone levels in fat were detected in *SDR9C7* and *RDH16* (Table S1). However, predictions based on the physical properties of these amino acid changes using SIFT (Kumar *et al*. 2009) do not suggest alterations in protein function. Three non‐synonymous SNPs were also found in the *tachykinin 3* (*TAC3*) gene located within the haplotype block at 24.05–24.56 Mb. *TAC3* is known to have a regulatory function in reproduction (Young *et al*. 2010), but these are not directly related to androstenone biosynthesis. Similar to the non‐synonymous SNPs in *SDR9C7* and *RDH16*, none of the three amino acid changes in *TAC3* suggest alterations in protein function. Two other genes within the block; *ENSSSCG00000026378*, at 23.9–24.0 Mb, and *ENSSSCG00000000413*, at 24.02–24.04 Mb, contain non‐synonymous SNPs predicted to having a deleterious effect on protein structure. These are, however, novel genes and their functions are not known.

This study confirms the presence of a QTL associated with androstenone levels in Norwegian Duroc pigs and narrows down the most likely position of this QTL to a haplotype block of 1.24 Mb at position 23.03–24.27 Mb on SSC5. The region is very gene rich and contains several putative candidate genes for androstenone biosynthesis. Due to extended LD within the region, we were unable to identify any single, explanatory causative variations, however information from this study can be used to reduce levels of androstenone in meat from entire males by implementing SNPs or haplotypes associated with lower levels of androstenone in breeding schemes. No associations were found with the sex steroids testosterone, estrone sulphate and 17*β*‐estradiol, suggesting that this can be done without perturbing levels of sex hormones essential for normal reproduction.

## Conflict of interest

The authors declare that they have no conflict of interest.

## Supporting information


**Table S1** Genotyped single nucleotide polymorphisms (SNPs).
