# Supplementary material for: Exploiting whole genome sequence data to fine map and characterize candidate genes within a quantitative trait loci region affecting androstenone on porcine chromosome 5
Source: Anim Genet. 2017 Oct 16;48(6):653–9. doi: 10.1111/age.12615 (PMC5698758; doi:10.1111/age.12615)
Supplement: Supplementary file 1 — Table S1 Genotyped single nucleotide polymorphisms (SNPs). [file AGE-48-653-s001.pdf]

Table S1 Genotyped single nucleotide polymorphisms (SNPs). The single nucleotide polymorphisms (SNPs) used for association analyses in Norwegian Duroc pigs. Their NCBI rs#, positions (pos) on *Sus scrofa10.2*, functional classes (class) and their effect, amino acid change (aa), genotyped minor allele frequency (MAF) values, percentage of explained variance, and log likelihood test (LRT) scores for androstenone (andr), testosterone (test), estrone sulphate (esul) and 17β-estradiol (estr) are given. An additional 21 SNPs from the Illumina 60K BeadChip were also included.

name	pos	andr	test	est	esul	MAF	variance	class	effect	aa	SIFT	Human associated gene name	effect	aa	gene name
rs322286479	22647977	17.72	0	0.025	0	0.232	4.5	Exon	Non-synonymous	R->W	0.04 (deleterious)	*RDH5*			
rs81383167	22648197	15.85	0	0.05	0	0.239	4	Exon	synonymous			*RDH5*			
rs344627572	22648284	18.10	0	0.025	0	0.233	4.5	Exon	synonymous			*RDH5*			
rs328425498	22648880	16.76	0	0.01	0	0.229	4.3	Intron				*RDH5*			
rs332798273	22649311	15.49	0	0.05	0	0.233	4	Intron				*RDH5*			
rs344314973	22651512	18.10	0	0.025	0	0.227	4.5	Intergenic							
rs345943585	22652372	9.69	0	0.01	0	0.324	2.7	Intron				*CD63*			
rs327655707	22673393	17.72	0	0.025	0	0.227	4.5	Intron				*GDF11*			
rs341310849	22689760	3.09	0	0	0	0.469	1.2	Intron				*SARNP*			
rs320775034	22702968	2.56	0	0	0	0.477	1.1	Intron				*SARNP*			
rs325280874	22719094	0.68	0	0	0	0.228	0.8	Intron				*SARNP*			
rs336011621	22741064	15.95	0	0.044	0	0.233	4.1	Intergenic							
rs320179428	22741496	17.72	0	0.043	0	0.230	4.5	Intergenic							
rs332386261	22752173	2.79	0	0	0	0.477	1.2	Exon	synonymous			*DNAJC14*			
rs339574266	22753153	15.95	0	0.044	0	0.234	4.1	Intron				*DNAJC14*			
rs319482986	22761452	0.53	0	0	0	0.239	0.8	Exon	synonymous			*MMP19*			
rs331600246	22762241	15.95	0	0.044	0	0.234	4.1	Exon	synonymous			*MMP19*			
rs341730558	22762778	15.95	0	0.044	0	0.231	4.1	Exon	synonymous			*MMP19*			
rs322122863	22765885	15.95	0	0.044	0	0.229	4.1	Exon	Non-synonym	G->R	0.18 (tolerat	*MMP19*			

									ous		ed low confid ence)			
rs324745822	22766002	15.95	0	0.044	0	0.234	4.1	Intergenic						
ASGA0025070	22773375	17.05	0	0.026	0	0.236	4.5	Intergenic						
rs330073140	22789156	16.11	0	0.105	0	0.142	4.8	Exon	3'UTR			*PYM1*		
rs340076148	22789517	16.35	0	0.026	0	0.234	4.1	Exon	3'UTR			*PYM1*		
rs80988884	22789895	16.35	0	0.026	0	0.232	4.1	Exon	3'UTR			*PYM1*		
rs332699091	22810938	16.35	0	0.034	0	0.234	4.1	Intergenic						
rs345932972	22811022	16.35	0	0.034	0	0.235	4.1	Intergenic						
rs324231280	22811366	16.35	0	0.034	0	0.237	4.1	Intergenic						
rs335090885	22811711	16.35	0	0.034	0	0.235	4.1	Exon	5'UTR			*DGKA*		
rs343055488	22817593	16.35	0	0.026	0	0.235	4.1	Exon	5'UTR			*DGKA*		
rs325193603	22822401	16.35	0	0.034	0	0.234	4.1	Exon	synonymous			*DGKA*		
rs336413988	22834572	1.73	0	0	0.05	0.281	1	Exon	synonymous			*DGKA*		
rs343221661	22837681	17.57	0	0.026	0	0.173	4.4	Intron				*PMEL*		
rs328542564	22889780	2.18	0	0	0	0.481	1	Intron				*PMEL*		
rs80977300	22900786	18.82	0	0.069	0	0.264	4.3	Intergenic						
rs80907514	22901225	3.73	0	0	0	0.451	1.3	Intergenic						
rs332584935	22912241	15.99	0	0.055	0	0.233	4.1	Intergenic						
rs342705026	22918137	0.68	0	0	0	0.235	0.8	Intergenic						
INRA0018914	22921968	17.05	0	0	0	0.236	4.5	Intergenic						
rs328001776	22922973	2.32	0	0	0	0.475	1	Intergenic						
rs196952176	22936766	16.35	0	0.055	0	0.234	4.1	Intergenic						
rs331501814	22940917	16.93	0	0.034	0	0.230	4.3	Intergenic						
DIAS0004735	22941207	2.35	0	0	0	0.481	1.1	Exon	5'UTR			*CDK2*		
rs318649076	22947123	15.81	0	0.072	0	0.290	4	Intergenic						

rs343410732	22948108	16.35	0	0.034	0	0.232	4.1	Intergenic							
rs327274312	22951109	16.35	0	0.055	0	0.232	4.1	Intergenic							
ASGA0025072	22969678	20.34	0	0	0	0.26	4.5	Intron				*ERBB3*			
rs329904578	22970594	18.82	0	0.069	0	0.259	4.3	Exon	5'UTR			*ERBB3*			
rs340905360	22980503	13.35	0	0	0	0.308	3.2	Intron				*ERBB3*			
rs324795185	22991309	6.08	0	0	0	0.496	1.7	Intron				*ESYT1, ERBB3*			
rs329190983	22992320	1.57	0	0	0.04	0.286	1	Exon	Non-synonymous	I->V	0.27 (tolerated)	*ESYT1*	Intron		*ERBB3*
rs337128796	23005094	1.55	0	0	0.03	0.286	1	Exon	3'UTR			*ESYT1*	Intron		*ERBB3*
rs343175926	23029661	0.69	0	0	0	0.181	0.8	Intron				*ERBB3*			
DIAS0001551	23032447	21.34	0	0	0	0.259	5.1	Intron				*SLC39A5, ERBB3*			
rs324131906	23032919	0.69	0	0	0	0.239	0.8	Exon	synonymous			*SLC39A5*	Intron		*ERBB3*
rs335374076	23035955	15.21	0	0	0	0.302	3.5	Intron				*SLC39A5, ERBB3*			
rs342097882	23042148	21.13	0	0.057	0	0.257	4.8	Intron				*NABP2, ERBB3*			
rs323397522	23043192	21.13	0	0.057	0	0.253	4.8	Exon	5'UTR			*NABP2*	Intron		*ERBB3*
rs334387135	23048314	4.29	0	0	0	0.452	1.4	Intron				*RNF41, ERBB3*			
rs344556303	23058166	21.13	0	0.081	0	0.255	4.8	Intron				*RNF41, ERBB3*			
ASGA0025077	23061869	21.13	0	0.05	0	0.259	4.8	Intron				*RNF41, ERBB3*			
ASGA0025075	23079108	21.34	0	0	0	0.259	5.1	Intron				*ERBB3*			
rs336528437	23087901	19.36	0	0.069	0	0.258	4.4	Exon	synonymous			*ERBB3*			
rs343826346	23120443	0.55	0	0	0	0.228	0.8	Intergenic							
rs327175412	23157363	4.36	0	0	0	0.460	1.5	Intron				*COQ10A*			
rs338250781	23172774	21.55	0	0.057	0	0.254	4.9	Intergenic							
rs318527203	23185783	22.11	0	0.057	0	0.207	5	Intergenic							
ASGA0025080	23190382	13.76	0	0	0	0.308	3.4	Intergenic							
rs321671602	23209605	0.58	0	0.056	0.02	0.194	0.8	Intergenic							

rs329932356	23246551	0.69	0	0	0	0.252	0.8	Intergenic							
rs80791879	23280756	21.13	0	0.081	0	0.254	4.8	Exon	synonymous			*STAT2*			
rs321408460	23283519	21.13	0	0.081	0	0.257	4.8	Intron				*STAT2*			
rs329107615	23289034	21.13	0	0.081	0	0.256	4.8	Intron				*STAT2*			
CADI0000251	23291033	21.13	0	0.081	0	0.259	4.8	Exon	synonymous			*STAT2*			
rs320695607	23296665	21.13	0	0.081	0	0.259	4.8	Exon	synonymous, splice region variant			*APOF*			
rs326008779	23308417	15.18	0	0	0	0.303	3.5	Exon	3'UTR			*TIMELESS*			
H3GA0016069	23313949	21.34	0	0	0	0.259	5.1	Intron				*TIMELESS*			
rs331819690	23332075	21.13	0	0.081	0	0.253	4.8	Intron				*TIMELESS*			
rs337190351	23333637	21.76	0	0.037	0	0.253	5	Intergenic							
rs45434332	23337793	21.13	0	0.037	0	0.256	4.8	Intron				*MIP*			
rs323351980	23501205	21.81	0	0.037	0	0.252	5	Intron				*RBMS2*			
rs334598159	23501329	21.81	0	0.037	0	0.257	5	Intron				*RBMS2*			
rs342746614	23506458	21.81	0	0.037	0	0.216	5	Intron				*RBMS2*			
rs324720234	23527868	18.15	0	0	0	0.335	3.8	Intergenic							
rs319134420	23605918	21.13	0	0.037	0	0.256	4.8	Intergenic							
rs333119909	23611111	21.13	0	0.037	0	0.251	4.8	Exon	3'UTR			*BAZ2A*			
rs45433675	23633645	21.13	0	0.037	0	0.250	4.8	Intron				*BAZ2A*			
rs330431342	23638649	21.13	0	0.037	0	0.253	4.8	Intergenic							
rs344431581	23643924	21.13	0	0.037	0	0.255	4.8	Intergenic							
ASGA0025083	23646360	25.75	0	0	0	0.284	5.4	Intergenic							
rs329732704	23652473	23.88	0	0	0	0.342	4.9	Intergenic							
rs55619179	23661024	21.13	0	0.035	0	0.235	4.8	Exon	synonymous			*ATP5B*			
ALGA0031261	23662089	21.13	0	0.035	0	0.262	4.8	Intron				*ATP5B*			

rs342473564	23664170	21.13	0	0.035	0	0.254	4.8	Intron				*ATP5B*			
rs339814725	23668458	22.10	0	0.019	0	0.217	5	Intergenic							
ALGA0031262	23686043	0	0	0	0	0.027	0	Intron				*PTGES3*			
rs326378603	23688504	21.13	0	0.121	0	0.257	4.8	Intron				*PTGES3*			
rs340409229	23695114	21.90	0	0.091	0	0.261	5	Intron				*PTGES3*			
rs324616431	23696268	21.55	0	0.121	0	0.246	4.9	Intergenic							
rs332357200	23708594	21.13	0	0.121	0	0.257	4.8	Intergenic							
H3GA0016074	23737420	21.34	0	0	0	0.259	5.1	Intergenic							
rs339985284	23755770	21.13	0	0.121	0	0.253	4.8	Intergenic							
rs324163538	23760884	21.13	0	0.121	0	0.250	4.8	Intergenic							
rs336052059	23767272	21.55	0	0.091	0	0.252	4.9	Intergenic							
rs321363767	23777965	21.13	0	0.121	0	0.256	4.8	Intron				*HSD17B6*			
rs325608908	23783769	21.13	0	0.121	0	0.256	4.8	Intron				*HSD17B6*			
rs339244595	23785359	21.13	0	0.121	0	0.254	4.8	Exon	synonymous			*HSD17B6*			
rs196952037	23785512	21.13	0	0.121	0	0.256	4.8	Exon	synonymous			*HSD17B6*			
rs331914905	23791879	21.13	0	0.121	0	0.254	4.8	Intron				*HSD17B6*			
rs343315339	23798333	21.13	0	0.121	0	0.264	4.8	Intergenic							
rs327169901	23798441	21.13	0	0.121	0	0.252	4.8	Intergenic							
rs338629716	23837331	21.13	0	0.121	0	0.258	4.8	Intergenic							
rs319779066	23838469	21.76	0	0.035	0	0.251	5	Intergenic							
rs331055272	23839270	21.29	0	0.102	0	0.245	4.8	Intergenic							
rs334482774	23894026	21.13	0	0.121	0	0.248	4.8	Intergenic							
rs344727562	23954716	0.014	0	0.083	0.36	0.225	0	Intergenic							
rs80913640	23965462	21.28	0	0.037	0	0.258	4.8	Exon	synonymous			*ENSSSCG00000026524*	synonymous		*STAT6*
rs322701353	23977771	21.69	0	0.022	0	0.255	4.9	Exon	synonymous			*ENSSSCG00000000411*			

rs334696417	23978441	21.28	0	0.037	0	0.259	4.8	Intron				*ENSSSCG00000000411*			
rs340206243	23978565	21.28	0	0.037	0	0.258	4.8	Intron				*ENSSSCG00000000411*			
rs323573852	23988945	21.28	0	0.037	0	0.224	4.8	Intergenic							
rs334799915	23989714	8.92	0	0	0	0.488	2.2	Intergenic							
ASGA0025092	23994070	21.49	0	0	0	0.265	5	Intron				*ENSSSCG00000026378*	Intron		*ENSSSCG00000025542*
rs341449423	24000917	20.32	0	0.037	0	0.257	4.6	Exon	synonymous			*ENSSSCG00000026378*	synonymous		*ENSSSCG00000025542*
rs326279394	24004105	8.22	0	0	0	0.489	2.1	Exon	Non-synonymous	T->M	0.26 (tolerated)	*ENSSSCG00000026378*	Non-synonymous	T->M	*ENSSSCG00000025542*
rs334619554	24006898	21.48	0	0.029	0	0.265	5	Exon	Non-synonymous	A->S	0.01 (deleterious)	*ENSSSCG00000026378*	Non-synonymous	A->S	*ENSSSCG00000025542*
rs325932073	24010913	7.27	0	0	0.07	0.462	1.9	Intergenic							
rs346272984	24020029	21.69	0	0.037	0	0.255	4.9	Intergenic							
rs325511177	24020038	0	0	0.168	0.32	0.185	0	Intergenic							
rs339156317	24029234	23.16	0	0.084	0	0.265	5.1	Intron				*ENSSSCG00000000413*	Intron		*ENSSSCG00000000424*
rs320473101	24040827	21.28	0	0.066	0	0.26	4.8	Exon	Non-synonymous	I->V	0.02 (deleterious)	*ENSSSCG00000000413*			
DIAS0004585	24041995	21.28	0	0.037	0	0.262	4.8	Exon	synonymous, splice region variant			*ENSSSCG00000000413*			
rs81312698	24045687	1.086	0	0	0	0.255	0.9	Intergenic							
rs320916955	24047518	21.28	0	0.037	0	0.254	4.8	Intergenic							
rs336038669	24049288	21.28	0	0.037	0	0.257	4.8	Exon	Non-synonymous	S->G	1 (tolerated)	*TAC3*			
rs319700230	24051985	21.28	0	0.037	0	0.259	4.8	Exon	Non-synonymous	H->R	1 (tolerated)	*TAC3*			
rs331868502	24053743	21.28	0	0.037	0	0.261	4.8	Exon	Non-synonymous	A->V	0.53 (tolerated)	*TAC3*			
rs328621205	24057597	22.24	0	0.015	0	0.223	5	Intergenic							
ASGA0103650	24057900	21.34	0	0	0	0.262	5.1	Intergenic							

rs339225409	24066320	21.55	0	0.057	0	0.257	4.9	Intergenic							
rs81475530	24067157	21.13	0	0.081	0	0.255	4.8	Exon	synonymous			*ZBTB39*			
rs196960371	24074908	21.13	0	0.081	0	0.257	4.8	Intergenic							
rs341893949	24087159	21.13	0	0.081	0	0.263	4.8	Intergenic							
rs323471701	24112111	21.13	0	0.121	0	0.256	4.8	Intron				*SDR9C7*			
rs331281514	24113102	22	0	0.121	0	0.255	5	Intron				*SDR9C7*			
rs338921529	24115262	22	0	0.05	0	0.256	5	Intron				*SDR9C7*			
rs322705134	24121188	21.68	0	0.03	0	0.259	4.9	Intron				*SDR9C7*			
rs334483880	24124376	21.76	0	0.081	0	0.254	5	Intron				*SDR9C7*			
rs346262620	24125051	21.13	0	0.081	0	0.256	4.8	Exon	Non-synonymous	G->S	1 (tolerated)	*SDR9C7*			
rs322041624	24125371	21.13	0	0.081	0	0.253	4.8	Intergenic							
rs340912662	24125786	21.13	0	0.081	0	0.261	4.8	Intergenic							
rs318348532	24126544	21.13	0	0.081	0	0.256	4.8	Intergenic							
rs329647381	24127715	21.13	0	0.081	0	0.256	4.8	Intergenic							
rs340590528	24129261	21.76	0	0.081	0	0.248	5	Intergenic							
rs320946775	24131309	21.13	0	0.081	0	0.260	4.8	Intergenic							
rs333429894	24134363	21.13	0	0.081	0	0.258	4.8	Intron				*RDH16*			
rs343546896	24136112	21.48	0	0.081	0	0.254	4.9	Exon	Non-synonymous	R->K	0.15 (tolerated)	*RDH16*			
rs328269823	24137957	21.13	0	0.081	0	0.310	4.8	Intron				*RDH16*			
rs335585083	24138240	21.76	0	0.19	0	0.258	5	Intron				*RDH16*			
rs329020205	24139064	21.28	0	0.084	0	0.259	4.8	Intergenic							
rs340508837	24139749	21.47	0	0.121	0	0.243	4.9	Intergenic							
rs324066652	24153921	0	0	0.337	0.61	0.230	0	Intergenic							
rs343644453	24208703	10.79	0	0	0	0.174	3.2	Intron				*STAT6*			
rs328095588	2421	21.69	0	0.022	0	0.258	4.9	Exon	synonym			*STAT6*			

	3673								ous						
rs339194671	24214827	21.28	0	0.037	0	0.259	4.8	Exon	synonymous			*STAT6*			
rs318931955	24221240	11.53	0.17x	1.554	0.62	0.323	2.2	Exon	3'UTR			*STAT6*			
rs331063564	24221247	0	0.14	1.046	1.35	0.163	0	Exon	3'UTR			*STAT6*			
rs337948009	24271893	21.28	0	0.037	0	0.259	4.8	Intron				*MYO1A*			
ALGA0031268	24293790	1.312	0	0	0	0.101	2	Intron				*LRP1*			
ALGA0031274	24330746	0	0	0	0	0.159	0	Intron				*LRP1*			
ASGA0025097	24354867	1.312	0	0	0	0.1	2	Intergenic							
rs321543967	24366112	0.402	0	0	0	0.209	0.7	Intergenic							
rs329517918	24383196	3.432	0	0	0	0.473	1.3	Exon	3'UTR			*STAC3*			
rs335963889	24390747	1.96	0	0	0	0.439	1	Intergenic							
rs338094154	24400567	4.16	0	0	0	0.385	1.5	Intron				*R3HDM2*			
rs339779477	24493471	4.458	0	0	0	0.388	1.6	Intron				*R3HDM2*			
rs323132897	24504464	18.52	0	0.003	0	0.256	4.3	Intron				*R3HDM2*			
rs322722625	24664102	4.598	0	0	0	0.387	1.6	Intergenic							
rs330474286	24709008	0	0	0	0	0.279	0	Intron							
H3GA0016090	24726597	18.70	0	0	0	0.345	4.3	Intron				*MARS*			
rs334122301	24776549	20.30	0	0.087	0	0.165	4.6	Intron				*MARS*			
rs81490455	24809910	0.258	0	0	0	0.073	0.9	Intron				*DTX3*			
rs333372477	24831917	16.47	0	0	0	0.328	3.4	Intergenic							
